# Supplementary material for: Detailed molecular and epigenetic characterization of the pig IPEC-J2 and chicken SL-29 cell lines
Source: iScience. 2023 Feb 20;26(3):106252. doi: 10.1016/j.isci.2023.106252 (PMC10018572; doi:10.1016/j.isci.2023.106252)
Supplement: Data S2. Complete homer output for identified motifs in Chicken SL-29, related to Tables 5 and 6 — Homer motif analysis results for histone modifications H3K4me1, H3K4me3, H3K27ac, enhancers, and ATAC-seq of chicken SL-29 cell line. Parameters for possible false positives is as mentioned earlier for S5. [file mmc3.zip › Data_S2/S6/Chicken_SL_29/peak_files_enhancers/homerResults/motif4.similar.html]

motif4

## Information for motif4

C
G
A
T
A
C
T
G
A
C
T
G
G
C
T
A
C
G
T
A
A
C
G
T
A
C
T
G
A
G
C
T
  
Reverse Opposite:  

C
T
G
A
G
T
A
C
C
G
T
A
A
C
G
T
C
G
A
T
A
G
T
C
A
G
T
C
C
G
T
A
  

|  |  |
| --- | --- |
| p-value: | 1e-41 |
| log p-value: | -9.444e+01 |
| Information Content per bp: | 1.839 |
| Number of Target Sequences with motif | 3114.0 |
| Percentage of Target Sequences with motif | 16.82% |
| Number of Background Sequences with motif | 4204.5 |
| Percentage of Background Sequences with motif | 13.33% |
| Average Position of motif in Targets | 154.1 +/- 83.5bp |
| Average Position of motif in Background | 150.0 +/- 86.9bp |
| Strand Bias (log2 ratio + to - strand density) | -0.1 |
| Multiplicity (# of sites on avg that occur together) | 1.11 |
| Motif File: | file (matrix) reverse opposite |

### Similar de novo motifs found

|  |  |  |  |  |  |  |  |
| --- | --- | --- | --- | --- | --- | --- | --- |
| Rank | Match Score | Redundant Motif | P-value | log P-value | % of Targets | % of Background | Motif file |
| 1 | 0.823 | T G A C G T C A T G A C C G T A A C G T A C G T G T A C A G T C A C G T C A T G | 1e-28 | -65.581970 | 6.83% | 4.96% | motif file (matrix) |
| 2 | 0.610 | G A C T G C A T T A G C A T C G G T A C A T C G T A C G T A G C | 1e-26 | -59.945745 | 57.98% | 54.09% | motif file (matrix) |
